# Genomic landscape of DNA repair genes in cancer

**DOI:** 10.18632/oncotarget.8196

**Published:** 2016-03-19

**Authors:** Young Kwang Chae, Jonathan F. Anker, Benedito A. Carneiro, Sunandana Chandra, Jason Kaplan, Aparna Kalyan, Cesar A. Santa-Maria, Leonidas C. Platanias, Francis J. Giles

**Affiliations:** ^1^ Northwestern Medicine Developmental Therapeutics Institute, Chicago, IL, USA; ^2^ Robert H. Lurie Comprehensive Cancer Center of Northwestern University, Chicago, IL, USA; ^3^ Northwestern University Feinberg School of Medicine, Chicago, IL, USA; ^4^ Division of Hematology-Oncology, Department of Medicine, Jesse Brown VA Medical Center, Chicago, IL, USA

**Keywords:** DNA repair, cancer, mutations, copy number variations, gene expression

## Abstract

DNA repair genes are frequently mutated in cancer, yet limited data exist regarding the overall genomic landscape and functional implications of these alterations in their entirety.  We created comprehensive lists of DNA repair genes and indirect caretakers.  Mutation, copy number variation (CNV), and expression frequencies of these genes were analyzed in COSMIC. Mutation co-occurrence, clinical outcomes, and mutation burden were analyzed in TCGA. We report the 20 genes most frequently with mutations (*n* > 19,689 tumor samples for each gene), CNVs (*n* > 1,556), or up- or down-regulated (*n* = 7,998).  Mutual exclusivity was observed as no genes displayed both high CNV gain and loss or high up- and down-regulation, and CNV gain and loss positively correlated with up- and down-regulation, respectively. Co-occurrence of mutations differed between cancers, and mutations in many DNA repair genes were associated with higher total mutation burden. Mutation and CNV frequencies offer insights into which genes may play tumor suppressive or oncogenic roles, such as *NEIL2* and *RRM2B*, respectively.  Mutual exclusivities within CNV and expression frequencies, and correlations between CNV and expression, support the functionality of these genomic alterations. This study provides comprehensive lists of candidate genes as potential biomarkers for genomic instability, novel therapeutic targets, or predictors of immunotherapy efficacy.

## INTRODUCTION

Defective DNA repair is a common hallmark of cancer. Cells are estimated to experience over 20,000 DNA damaging events each day [1], which are normally repaired by specific DNA repair pathways with no lasting effects. The base excision repair (BER) pathway is responsible for sensing and repairing single-strand breaks (SSBs) in DNA while the homologous recombination (HR) and non-homologous end joining (NHEJ) pathways heal double-strand breaks. The mismatch repair (MMR) pathway corrects for inappropriate nucleotide insertions, deletions, and single nucleotide mismatched incorporations. Additionally, the nucleotide excision repair (NER) pathway corrects ultraviolet radiation-induced pyrimidine dimers and other helix-distorting lesions. The Fanconi anemia (FA) pathway consists of a core complex that recognizes interstrand crosslinks (ICLs), a multi-subunit ubiquitin ligase, and downstream repair nucleases. And the direct reversal (DR) pathway removes damaging DNA methylations *via* O^6^-methylguanine DNA methyltransferase (MGMT) [2, 3]. Aberrations in genes involved in these pathways are closely linked to the development of malignancies. Inactivating mutations and hypermethylation in MMR genes (i.e. *MSH2, MSH6, MLH1, PMS1,* and *PMS2)* lead to the development of Lynch syndrome and microsatellite instability (MSI), conferring a 70% lifetime risk of colorectal cancer (CRC) and an increased risk of developing other cancers [4]. Germline mutations in *BRCA1* and *BRCA2,* involved in HR and FA repair, increase the risk of developing breast and ovarian cancer 40-80% and 11-40%, respectively, in addition to other cancer types [5]. Additional links include defects in the HR gene, *ATM,* being associated with ataxia telangiectasia and up to a 25% lifetime malignancy risk [6], while NER defects in xeroderma pigmentosum are associated with a 70% risk of skin cancer by 8 years of age [7].

However, it remains unclear whether all DNA repair mutations are truly causal in driving tumorigenesis, as “mountain” genes, or are a byproduct of the malignancy and represent more infrequently mutated “hills” [8]. In support of the former is the ‘mutator phenotype’ and the concept that early mutations in critical genes, such as those involved in DNA repair, result in genomic instability and subsequent hypermutability, accounting for the high mutation rate seen in cancer [9]. This theory of causality is further supported by reports that MMR mutations and MSI are commonly seen in early adenomas and early stage CRC [10, 11].

Despite the uncertainty of whether all defects in the DNA damage response play a central role in cancer pathogenesis, they are highly significant in disease progression and treatment. Several studies have linked upregulation of DNA repair genes with chemo- and radio-resistance in multiple tumor types [12] and with the ability of tumors to metastasize [13, 14]. Therefore, while loss of DNA repair function is significant in cancer initiation, gain of function of similar genes and re-activation of lost repair pathways is involved in disease progression [15, 16]. This may explain why patients with muscle-invasive bladder cancer carrying at least one somatic mutation in a critical DNA repair gene had increased recurrence-free survival compared to patients without these mutations [17]. In addition, targetable DNA repair inhibition has also been shown to enhance tumor responses. PARP1, involved in the BER pathway through its ability to sense and repair SSB lesions *via* ADP-ribosylation, has become a successful therapeutic target [18]. Olaparib, a PARP1 inhibitor, is now approved for patients with advanced ovarian cancer harboring *BRCA1* or *BRCA2* deleterious mutations, as loss of *BRCA* sensitizes these tumors to further inhibition of DNA repair and results in a synthetic lethality [19, 20]. PAPR1 inhibitors also show potential in many other cancers harboring deficiencies in DNA repair [21], and inhibition of other DNA repair genes is being evaluated to induce synthetic lethality, including PRKDC inhibition in *MYC*-overexpressing tumors [22]. Additionally, the emerging field of personalized immunotherapies directed specifically against mutated cancer ‘neo-antigens’ may ultimately prove to be strongly linked to impairment in DNA repair [23].

Even with our knowledge about the genes involved in the multiple pathways of the DNA damage response, few studies have evaluated the genomic landscape of all DNA repair genes in its entirety on a large scale in cancer. A recent study analyzing the mutational landscape and copy number variation (CNV) alterations in 100 pancreatic tumors revealed a strong association between genomic instability and inactivation of DNA repair genes, and was able to identify new candidate genes in driving pancreatic tumorigenesis [24]. In this study, we analyzed a comprehensive list of DNA repair genes utilizing the large databases Catalogue Of Somatic Mutations In Cancer (COSMIC) [25] and The Cancer Genome Atlas (TCGA) within cBioPortal [26, 27]. These genes were analyzed in all cancer types (in 19,689 to 97,717 tumor samples for each gene in COSMIC) as well as in specific cancer types. Genes were further classified as direct components of DNA repair (directly involved in at least one of MMR, HR, FA, NER, BER, NHEJ, or DR) or as caretaker genes indirectly involved in maintaining genomic stability (Supp. Table 1).

## RESULTS

We analyzed a total of 193 DNA repair genes; 122 were considered directly involved in at least 1 DNA repair pathway and 71 were classified as caretaker genes indirectly involved in maintaining genomic stability (Supp. Table 1). These genes were evaluated for mutation frequency in sequenced tumor samples from the COSMIC database for all combined cancer types (19,689 to 97,717 unique tumor samples for each gene), as well as lung (1,593 to 7,681 samples per gene), breast (1,265 to 11,869 samples), liver (1,270 to 4,177 samples), large intestine (LI; 86.9% CRC excluding tumors of non-specific sub-tissue localization; 1,052 to 13,101 samples), and skin (92.5% melanoma; 805 to 3,353 samples) cancers. The top 20 most frequently mutated genes in each group are listed in Table 1. *TP53* was the most frequently mutated gene in all evaluated cancer types (26.9% of all cancers, 22.9%-43.9% in specific cancer types), while *MLL3, ATM, PRKDC, BRCA2, POLQ, ATR, POLE,* and *REV3L* were also within the top 20 list of all 6 groups, *BRCA1* and *FANCM* in the top 20 for 5 of the 6 groups, and *CENPE, SLX4, CDK12, SHPRH,* and *FANCD2* within the top 20 most frequently mutated DNA repair genes in 4 of the 6 evaluated groups. There were also genes frequently mutated specifically in 1 or 2 of the evaluated groups, including *MSH4, MDC1, WRN,* and *LIG1* in lung cancer, *PALB2, BLM,* and *FANCA* in breast cancer, *RAD50, MSH3, MLH3, BLM, CLK2,* and *ERCC2* in liver tumors, *MLH1, LIG1, TTK,* and *MSH2* in LI cancer, and *MDC1* and *POLD1* in skin cancer.

**Table 1:** Frequently mutated DNA repair genes in common cancers

All	%	Lung	%	Breast	%	Liver	%	LI	%	Skin	%
*TP53*	27.5	*TP53*	33.7	*TP53*	22.9	*TP53*	26.6	*TP53*	43.9	*TP53*	24.7
*MLL3*	5.2	*MLL3*	9.1	*MLL3*	7.1	*MLL3*	3.3	***ATM***	19.7	*MLL3*	13.5
***ATM***	4.8	***ATM***	4.5	***ATM***	2.2	***ATM***	3.2	*MLL3*	13.2	*POLQ*	8.1
*BAP1*	2.5	*POLQ*	4.3	***BRCA1***	1.9	***PRKDC***	1.9	***PRKDC***	12.1	***ATM***	7.2
***BRCA2***	2.5	***PRKDC***	4.0	*CENPE*	1.9	***ATR***	1.8	***BRCA2***	11.2	***ATR***	6.6
*POLQ*	2.1	***FANCM***	3.2	***BRCA2***	1.6	***BRCA2***	1.7	***MSH6***	11.0	***BRCA2***	6.4
***PRKDC***	2.1	*CENPE*	2.8	*TP53BP1*	1.6	*POLQ*	1.5	***MLH1***	8.8	***SLX4***	6.3
***ATR***	1.8	***BRCA2***	2.8	***PRKDC***	1.5	*REV3L*	1.3	*POLQ*	8.7	*TP53BP1*	6.3
***BRCA1***	1.8	*SHPRH*	2.4	***FANCD2***	1.4	***POLE***	1.3	*REV3L*	8.2	***PRKDC***	5.5
***MSH6***	1.8	***ATR***	2.4	***ATR***	1.4	*KNTC1*	1.3	***ATR***	8.2	*CENPE*	5.1
***POLE***	1.7	***EXO1***	2.4	***POLE***	1.3	***MSH6***	1.0	***LIG1***	7.7	*MDC1*	5.0
*REV3L*	1.7	***MSH4***	2.4	*POLQ*	1.2	***RAD50***	1.0	*TTK*	7.7	*REV3L*	4.7
***FANCM***	1.6	***POLE***	2.3	***ERCC6***	1.1	***MSH3***	0.9	***POLE***	7.5	***POLE***	4.5
*CENPE*	1.6	*MDC1*	2.3	*REV3L*	1.1	***MLH3***	0.9	***BRCA1***	7.3	***BRCA1***	4.4
*TP53BP1*	1.4	*WRN*	2.1	*CDK12*	1.0	*BLM*	0.9	***MSH2***	7.2	***FANCM***	4.1
*CDK12*	1.3	***BRCA1***	2.1	*TOPBP1*	1.0	*CLK2*	0.9	***SLX4***	6.9	*BAP1*	4.0
***FANCD2***	1.3	*REV3L*	2.0	***PALB2***	1.0	*BAP1*	0.9	*SHPRH*	6.3	***POLD1***	4.0
*SHPRH*	1.3	*CDK12*	1.8	*SHPRH*	0.9	***FANCM***	0.9	***FANCM***	6.3	***FANCD2***	3.9
*KNTC1*	1.3	***ERCC6***	1.8	*BLM*	0.9	***ERCC2***	0.9	***FANCD2***	6.2	***ERCC6***	3.9
***SLX4***	1.3	***LIG1***	1.8	***FANCA***	0.9	***SLX4***	0.9	*CDK12*	6.2	*KNTC1*	3.7

Top 20 most frequently mutated DNA repair genes in all combined cancers, lung, breast, liver, large intestine (LI; 86.9% colorectal cancer), and skin cancer (92.5% melanoma) from COSMIC. Bold = direct DNA repair genes, non-bold = caretaker genes indirectly involved in genomic stability, blue = potential oncogenes with high CNV gain frequencies (Figure 2A), red = potential tumor suppressors with high CNV loss frequencies (Figure 2A).

**Figure 1:**
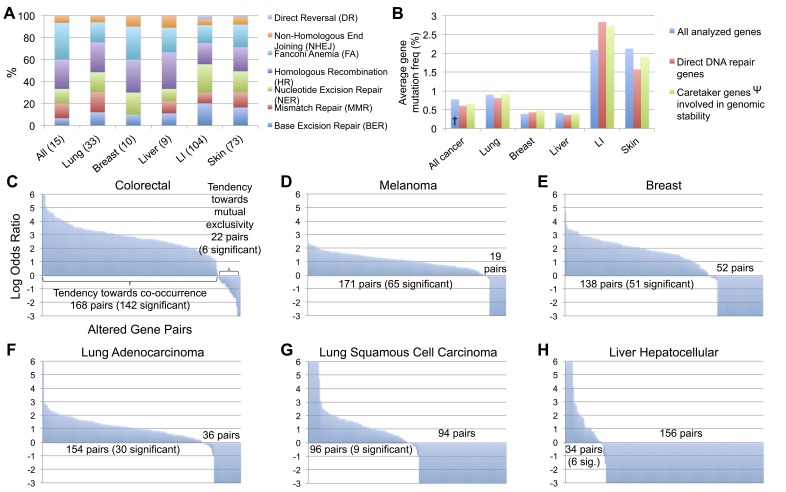
Analysis of DNA repair mutations by repair pathway, direct or indirect classification, and co-occurrence or mutual exclusivity between tumors **A.** Breakdown by pathway for direct DNA repair genes mutated in > 1% of each cancer type (number of included genes in parentheses). **B.** Average gene mutation frequency for all COSMIC analyzed genes, direct DNA repair genes, and indirect genomic stability maintenance genes by cancer type. **C.**-**H.** Tendency toward co-occurrence or mutual exclusivity for all possible 190 pairs of the top 20 most frequently mutated DNA repair genes from Table 1 for each cancer types, analyzed in TCGA. † All analyzed genes in All cancer excludes cancer types with < 200 sequenced tumors or < 1000 analyzed genes. Ψ Values for Caretaker genes involved in genomic stability excludes *TP53.*

Each cancer type was then analyzed for the degree to which the 7 DNA repair pathways were affected, when considering the number of direct DNA repair genes with a mutation frequency greater than 1%. Relative to the aberrations in all combined cancer types, lung cancer appeared to be strongly impacted in BER and HR, breast cancer in FA and HR, liver cancer in MMR and HR, LI cancer in BER, MMR, and NER, and skin cancer in BER, NER, and the FA repair pathway (Figure 1A). When comparing all COSMIC analyzed genes, direct DNA repair genes, and indirectly involved repair genes (excluding *TP53*), the mutation frequencies were consistent among the three groups within all combined cancers (consisting of 22 cancer types, each with > 200 samples and > 1000 genes analyzed), lung, breast, liver, and skin cancer. LI and skin cancer were the most heavily mutated cancers for all analyzed genes, in agreement with previous reports [8, 35], with LI displaying a further 0.74% and 0.64% increased mutation frequency of direct and indirect DNA repair genes, respectively, in comparison to the LI cancer average mutation frequency of all COSMIC genes (Figure 1B). Additionally, the degree to which these mutations co-occurred within samples or were mutually exclusive also differed between tumor types. Of the 20 most frequently mutated genes in each cancer type (Table 1), all possible 190 gene pairs were analyzed within TCGA. CRC, melanoma, breast, and lung adenocarcinoma displayed a strong tendency toward mutated gene pairs co-occurring within tumors (Figure 1C-1F). Lung squamous cell carcinoma samples displayed 94 genes pairs with a tendency toward mutual exclusivity (none significant) and 96 gene pairs toward co-occurrence (9 significant) (Figure 1G), while the majority of mutated gene pairs (156) in liver hepatocellular carcinoma samples were trending towards mutual exclusivity (0 significant, 9 gene pairs significant for co-occurrence) (Figure 1H).

**Figure 2:**
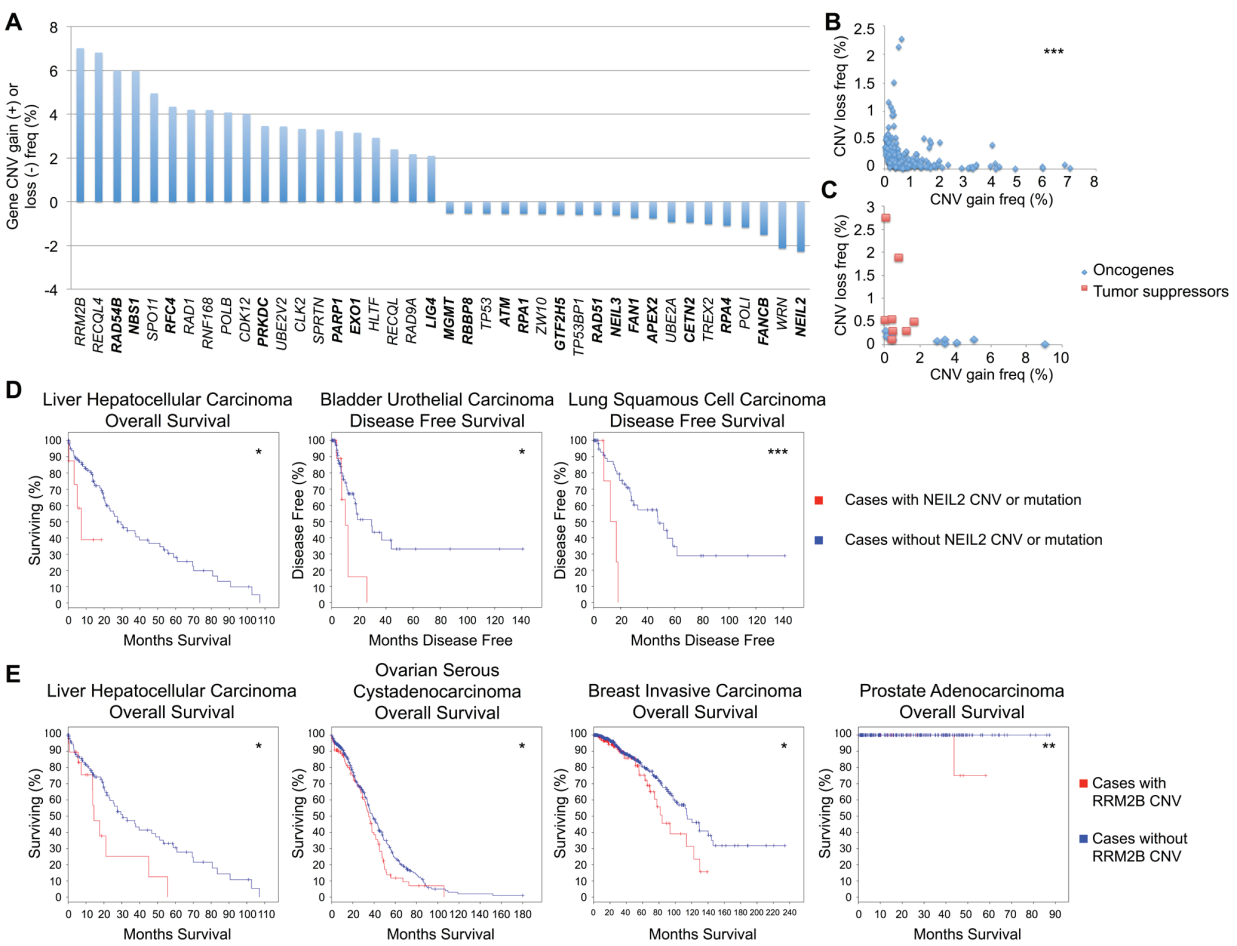
DNA repair gene CNV frequencies **A.** Top 20 DNA repair genes most frequently with CNV gain or loss, analyzed in COSMIC. Bold = direct DNA repair genes, non-bold = caretaker genes indirectly involved in genomic stability. **B.** Mutual exclusivity of CNV gain and loss within each DNA repair gene (*r* = −0.285). **C.** Mutual exclusivity of CNV gain and loss within common breast cancer oncogenes and tumor suppressors (*r* = −0.335), analyzed in COSMIC. **D.** Overall survival and disease free survival curves for cases with and without CNV or mutations in NEIL2, from TCGA. **E.** Overall survival curves for cases with and without RRM2B CNV, from TCGA. *p < 0.05, **p < 0.01, ***p < 0.001.

The gene list was further genomically analyzed for frequencies of CNV gain or loss within all combined cancer types. The top 20 genes most frequently displaying CNV gain or loss are displayed in Figure 2A. *RRM2B, RECQL4, RAD54B,* and *NBN* were the most commonly amplified genes and *NEIL2, WRN, FANCB,* and *POLI* most frequently displayed copy number loss. A highly statistically significant mutual exclusivity was observed when comparing the CNV gain and loss frequencies of these genes (Figure 2B). This trend was also observed when plotting out the CNV gain and loss frequencies of well known breast cancer oncogenes and tumor suppressors [36], with the former displaying predominantly CNV gain and the latter primarily CNV loss (Figure 2C).

To validate this relationship between DNA repair genes and potential oncogenic or tumor suppressive roles, we utilized the TCGA database to analyze *NEIL2* and *RRM2B*, the repair genes with the highest frequency of CNV loss and gain, respectively. Cases with mutations (assuming loss of function) or CNV alterations (all CNV loss, other than 1 CNV gain lung cancer case) in *NEIL2* displayed decreased OS in liver hepatocellular carcinoma and decreased DFS in bladder urothelial and lung squamous cell carcinoma (Figure 2D). Cases with CNV alterations (all CNV gain, other than 1 CNV loss liver cancer case and 1 CNV loss breast cancer case) in *RRM2B* were associated with decreased OS in liver, breast, and prostate carcinoma, and ovarian serous cystadenocarcinoma (Figure 2E). The changes in OS and DFS for these and other tumor types not shown were not significant. However, bladder cancer with *NEIL2* CNV alterations and mutations was trending toward decreased OS, and those with *RRM2B* CNV alterations showed a non-significant decrease in OS in colorectal, esophageal, and head and neck squamous cell carcinoma and a non-significant decreased DFS in bladder, lung adenocarcinoma, and CRC (data not shown). Potential oncogenes with high CNV gain also present in the top 20 most frequently mutated DNA repair genes (Table 1) include *CDK12*, *PRKDC*, *CLK2*, and *EXO1,* while potential tumor suppressors with high CNV loss found in Table 1 include *TP53, ATM, TP53BP1,* and *WRN.*

**Figure 3:**
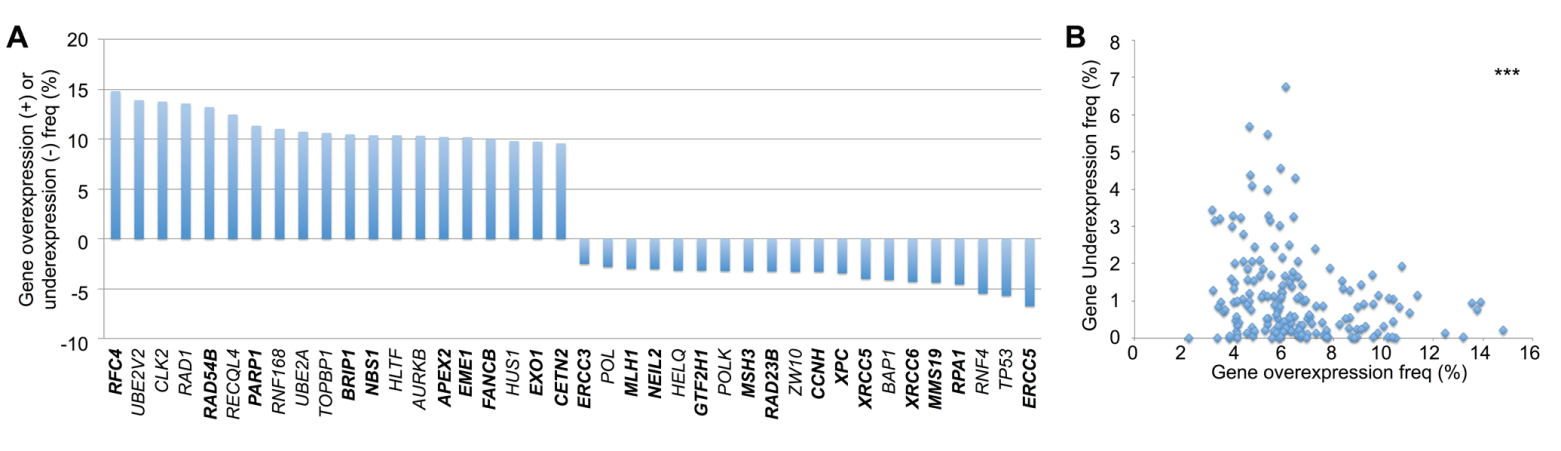
DNA repair gene up- and down-regulation frequencies **A.** Top 20 DNA repair genes most frequently overexpressed or underexpressed, analyzed in COSMIC. Bold = direct DNA repair genes, non-bold = caretaker genes indirectly involved in genomic stability. **B.** Mutual exclusivity of overexpression and underexpression within each gene (*r* = −0.249). ***p < 0.001.

A similar analysis was performed to plot the top 20 genes most frequently displaying overexpression or underexpression in tumor samples (Figure 3A). *RFC, UBE2V2, CLK2,* and *RAD1* were most commonly overexpressed, while *ERCC5, TP53, RNF4,* and *RPA1* were the most frequently underexpressed DNA repair genes. A similar statistically significant trend was observed as in the CNV data, revealing a mutual exclusivity in that no single gene displayed a high frequency of both overexpression and underexpression (Figure 3B).

**Figure 4:**
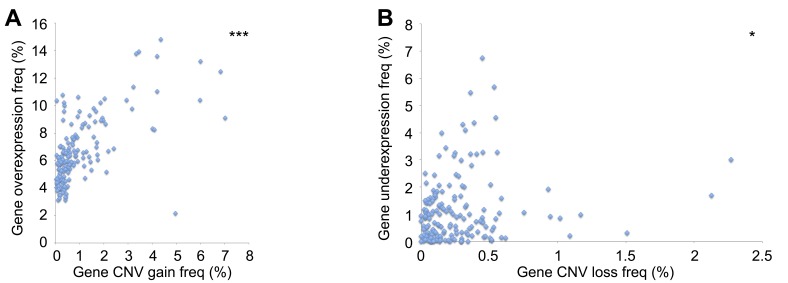
Association between DNA repair CNV and gene expression **A.** Positive correlation between CNV gain and gene overexpression (*r* = 0.632). **B.** Positive correlation between CNV loss and gene underexpression (*r* = 0.178). *p < 0.05, ***p < 0.001.

To provide support for whether the genomic alterations we observed were functional within these tumors, we compared gene overexpression with CNV gain data, as well as gene underexpression with CNV loss. Both analyses revealed a statically significant positive correlation (Figure 4A, 4B).

**Figure 5:**
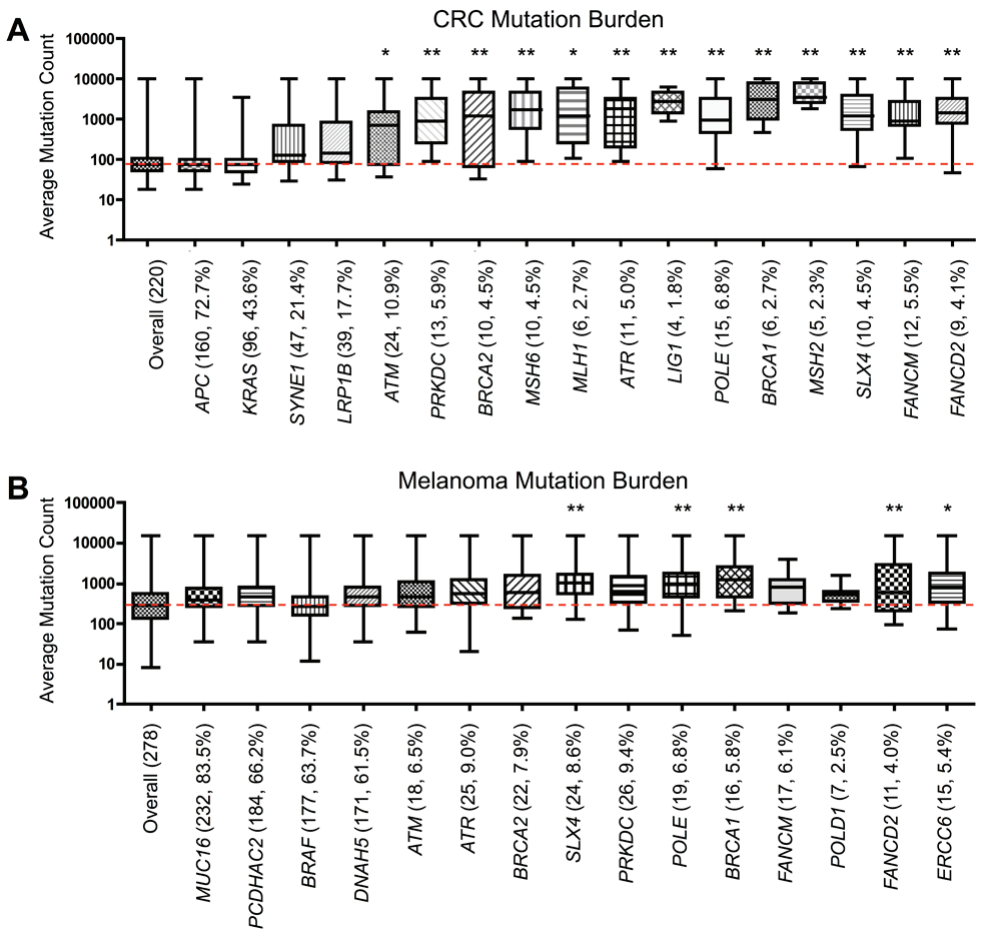
Somatic mutational burden in tumors with DNA repair gene mutations Average **A.** colorectal and **B.** melanoma tumor mutation burden in samples containing a mutation within specific control genes or the most frequently mutated direct DNA repair genes from Table 1, analyzed in TCGA. Number of samples containing mutations in each gene and the percentage of samples containing a mutation (out of 220 for CRC, 278 for melanoma) included in parentheses. Red line represents the value for the average mutation count overall in CRC or melanoma. *p < 0.05, **p < 0.01, as determined by one-way ANOVA with post-hoc Dunnett analysis.

To determine if the DNA repair genes analyzed in this study were linked to a higher mutational burden, as hypothesized in the ‘mutator phenotype’ [9], we quantified the average mutation count from the populations of tumors containing mutations in individual DNA repair genes. CRC cases with mutations in any of the 13 most frequently mutated direct DNA repair genes in LI tumors (Table 1) contained a significantly increased average mutation burden relative to the average mutation count in all CRC samples (Figure 5A). In comparison, the 4 most frequently mutated non-DNA repair genes in CRC (*APC, KRAS, SYNE1, LRP1B*) did not contain an increased mutational burden. A similar trend was seen in melanoma, within *SLX4, POLE, BRCA1, FANCD2*, and *ERCC6*-mutated tumors containing a significantly higher mutational burden than overall melanoma (Figure 5B).

## DISCUSSION

Our study utilized the power of the large COSMIC and TCGA databases to determine the genomic landscape of DNA repair genes in their entirety, elucidating similarities and differences between multiple cancer types. While previous studies have identified specific DNA repair pathways enriched in different cancers, such as MMR in colorectal cancer and HR in breast cancer [3], here we report the degree to which all 7 DNA repair pathways are impacted in multiple cancer types. Lung, breast, liver, LI, and skin cancers each revealed unique patterns of aberrations in DNA repair pathways. Breast cancer displayed many mutations in HR and FA genes, LI cancer in BER, MMR, and NER, lung cancer in BER and HR, liver cancer in MMR and HR, and skin cancer in BER, NER, and FA pathway genes. Cancer types also differed in the degree to which mutations in DNA repair genes co-occurred or displayed mutual exclusivity in tumors, with CRC, melanoma, breast cancer, and lung adenocarcinoma trending toward the former and liver cancer heavily trending towards the latter. In addition, the average mutation frequencies of direct and indirect DNA repair genes were quantified. These data not only revealed the large increase in mutation frequency in LI cancers, but also provided specific values that can be of great use in future clinical trial design. These values, which have not before been documented in this manner, can aid in estimating the number of DNA repair mutations to be expected within specific cancer populations when designing cohort sizes.

This study has compiled and analyzed a comprehensive list of DNA repair genes that may serve as potential biomarkers of malignancies or as therapeutic targets. Genes were individually analyzed for frequency of mutations, CNVs, and expression level alterations. The mutual exclusivity seen with no genes displaying both high CNV gain and loss, as well as the mutual exclusivity between high gene upregulation and downregulation, serve to support the functionality of the observed genetic changes. Further endorsing the functional implications of our findings is the significant positive correlations between CNV gain and gene upregulation as well as CNV loss and gene downregulation. Interestingly, when plotting CNVs in commonly altered breast cancer genes, oncogenes displayed predominantly CNV gain and tumor suppressors CNV loss. A similar pattern was seen with CNVs in DNA repair genes, suggesting that many of these genes should be evaluated with a new perspective, as potential tumor suppressors whose loss cause genomic instability and a tumorigenic mutator phenotype, or as upregulated compensatory genes providing resistance to DNA damaging therapies or enhancing the ability to metastasize. This was validated with *NEIL2* and *RRM2B*, the genes most frequently displaying CNV loss and gain, respectively. CNVs in these genes were significantly associated with decreased OS and DFS in multiple tumor types, although it is not clear whether the clinical association seen was solely due to the effect of CNV gain or loss of the analyzed genes themselves or due to gain or loss of copy number of neighboring genes.

Interestingly, a previous study analyzing oncogenic signatures across 12 tumor types identified an inverse correlation between high rates of recurrent mutations and recurrent copy number alterations (including CNV gain and loss), with no tumors displaying a large number of both [37]. This is in agreement with our data, as only 4 of the top 20 most mutated DNA repair genes (excluding *TP53*) were also found within the 20 genes most frequently with CNV gain (*CDK12, PRKDC*) or the top 20 with CNV loss (*ATM, TP53BP1*). High rates of mutations or CNV alterations appear to be mutually exclusive phenomena, termed genome hyperbola [37], between genes in multiple tumor types.

A new, promising avenue in personalized medicine has been the identification of immunogenic tumor-mutated peptides, termed neo-antigens, that are expressed specifically from the tumor mutanome and may act as potential targets to predict or enhance the efficacy of immunotherapies [23]. In the murine melanoma cell line B16F10, multiple somatic mutations were identified, the immunogenicity of the resulting peptides were confirmed, and immunizing mice against these neo-antigens provided strong therapeutic value [38]. In multiple clinical histologies, mutations predicted to be immunogenic were associated with increased *CD8A* expression and patient survival [39]. Additionally, in melanoma patients who received adoptively transferred T cell therapy, tumor infiltrating lymphocytes (TILs) identified as recognizing candidate mutated peptides, as predicted by whole-exome sequencing and a major histocompatibility complex (MHC)-binding algorithm, were associated with tumor regression [40]. This approach has translated clinically, as a patient with metastatic cholangiocarcinoma displayed tumor regression after adoptive transfer therapy of CD4+ TILs specific for a mutated epitope identified during tumor sequencing [41].

When considering the efficacy of immune checkpoint inhibitors, promising clinical responses have been observed with anti-PD-1 and anti-CTLA-4 blockade in lung cancer and melanoma [42-45], both of which are associated with the highest cancer mutation rates [8, 35]. Candidate neo-antigens have already been identified and validated in ipilimumab and tremelimumab treated melanoma patients [46]. In pembrolizumab treated non-small cell lung cancers, higher burden of nonsynonymous mutations was associated with increased clinical response and progression-free survival (PFS), and responders with the greatest number of mutations contained mutations in the DNA repair genes *POLD1, POLE, MSH2, PRKDC, RAD17, BRCA2, RAD51C,* and/or *LIG3.* Also within this study, clinical response and increased PFS correlated with a higher burden of identified candidate neo-antigens, and a neo-antigen-specific T cell population was identified in one responder that correlated with tumor regression [47].

In muscle-invasive bladder cancer, mutations in the DNA repair genes including *ATM, ERCC2, FANCD2, PALB2, BRCA1*, or *BRCA2* were associated with higher somatic mutation burden, as measured by nonsynonymous single nucleotide variants and higher T cell clonality (a lower T cell receptor diversity index) [17, 48]. This hypermutable state generated by defects in the DNA repair mechanism may be responsible for altering the tumor mutanome, resulting in the production of highly tumor-specific mutated neo-antigens that may have contributed to the favorable recurrence-free survival after cystectomy in bladder cancer patients with DNA repair gene mutations. Similar phenomenon has also been observed in ovarian cancer. Tumors with germline or somatic mutation in *BRCA1/2* that harbored higher overall somatic mutational burden were associated with a much favorable overall prognosis compared with tumors with lower somatic mutational burden [49].

A recent phase 2 study of pembrolizumab in MMR deficient tumors examined the association between MMR deficiency status and the response to PD-1 blockade [50]. Pembrolizumab was administered to three different groups of patients with MMR-deficient colorectal cancers, MMR-proficient colorectal cancers, and MMR-deficient non-colorectal cancers. The immune-related objective response rate was 40% (95% CI: 12, 74) in MMR-deficient colorectal cancers, 0% (95% CI: 0, 19) in MMR-proficient colorectal cancers, and 71% (95% CI: 29, 96) in MMR-deficient non-colorectal cancers. Results from survival analyses favored MMR-deficient colorectal cancers compared to MMR-proficient colorectal cancers in terms of disease progression (*p* < 0.001) or death (*p* = 0.05). Whole-exome sequencing also revealed significant differences in mean somatic mutations per tumor between MMR-deficient tumors and MMR-proficient tumors (1782 vs. 73, *p* = 0.007). This study, for the first time, prospectively validated a significant association between DNA repair defects and response to therapy. It suggests that MMR status may be a strong predictor of clinical benefit from immune checkpoint blockade. The use of MMR status, and potential alterations in other DNA repair genes, may become increasingly valuable for predicting immunotherapeutic efficacy, as highlighted by the many issues with utilizing PD-L1 as a biomarker for patients receiving PD-1/PD-L1 pathway blockade [51].

In accordance with the above findings, we determined that many of these DNA repair mutations were significantly associated with a higher somatic mutational burden in cancer, including CRC and melanoma. Our study provides important insight into the genomic landscape of DNA repair genes in cancer on a large scale, and the resulting hypermutable state in affected tumors may prove to enhance the efficacy of future cellular and immune checkpoint targeted immunotherapies. The results from our study will help increase our knowledge of DNA repair genes, enabling us to design novel biomarker-based basket clinical trials across various tumor types that utilize both synthetic lethality and immune modulation.

## MATERIALS AND METHODS

A comprehensive list of genes involved in DNA repair was created from a literature review of previous publications on DNA repair genes in cancer. This gene list was cross-referenced with the list of commonly mutated cancer genes clinically evaluated in the FoundationOne™ platform (Foundation Medicine Incorporated, Cambridge, Massachusetts) [2, 3, 28-34]. Genes were then subclassified as directly-involved DNA repair genes and were divided into their associated DNA repair pathway or pathways, or were instead subclassified as indirect components of the DNA repair process involved in maintaining genomic stability (Supp. Table 1). Within the COSMIC database (cancer.sanger.ac.uk), each gene was analyzed for frequency of somatic mutations (in all cancer types and specifically within sequenced lung, breast, liver, large intestine large intestine (LI; 86.9% of samples were CRC), or skin tumors), CNV gain, CNV loss, overexpression, and underexpression. Using somatic nonsynonymous mutation data from the TCGA Research Network (http://cancergenome.nih.gov/) for several cancer types (LI tumor samples were 100% CRC) within cBioPortal for Cancer Genomics (http://www.cbioportal.org), DNA repair genes were analyzed for mutation co-occurrence or mutual exclusivity, their association with overall survival (OS) and disease free survival (DFS), and their association with total mutation burden.

## SUPPLEMENTARY MATERIAL TABLE


