# Supplementary material for: Genomic landscape of DNA repair genes in cancer
Source: Oncotarget. 2016 Mar 19;7(17):23312–21. doi: 10.18632/oncotarget.8196 (PMC5029628; doi:10.18632/oncotarget.8196)
Supplement: Supplementary file 1 [file oncotarget-07-23312-s001.pdf]

Genomic landscape of DNA repair genes in cancer

Supplementary Material

Supplemental Table 1: List of all 193 DNA repair genes analyzed. Classified as direct DNA repair genes or indirect regulators of genomic stability. Direct DNA repair genes further classified by involved repair pathway or pathways (1=BER, 2=MMR, 3=NER, 4=HR, 5=FA, 6=NHEJ, 7=DR).

**Direct DNA repair genes**	**DNA repair pathways**
ATM	4
ATR	5
RPA1	3, 4
RPA2	3, 4
RPA3	3, 4
RPA4	3, 4
BRCA1	4
BRCA2	4, 5
RAD51	4
RFC1	1, 2, 3
RFC2	1, 2, 3
RFC3	1, 2, 3
RFC4	1, 2, 3
RFC5	1, 2, 3
XRCC1	1
PCNA	1, 2, 3
PARP1	1
ERCC1	3, 5
MSH3	2
PMS2	2
MLH1	2
MSH6	2
MSH2	2

MLH3	2
EXO1	2
NBN	4
RAD50	4
CHEK2	4
FANCI	5
FANCD2	5
FANCA	5
FANCC	5
FANCE	5
FANCL	5
FANCG	5
FANCM	5
ERCC4	3
ERCC2	3
ERCC5	3
PARP2	1
APEX1	1
FEN1	1
XPC	3
ERCC6	3
GTF2H2	3
ERCC3	3
XPA	3
RAD23B	3
PALB2	4, 5
RAD51C	4, 5
XRCC6	6

XRCC5	6
PRKDC	6
XRCC4	6
Lig4	6
FANCB	5
FANCF	5
FAAP24	5
CHEK1	5
BRIP1	5
SLX4	5
FAN1	5
MUS81	4, 5
EME1	4, 5
POLE	1, 3
POLD1	1, 2, 3
MRE11A	4
RAD51D	4
RAD52	4
RAD51B	4
PMS1	2
RAD23A	3
LIG3	1, 3
MGMT	7
OGG1	1
UNG	1
SMUG1	1
MBD4	1
TDG	1

MUTYH	1
NTHL1	1
MPG	1
NEIL1	1
NEIL2	1
NEIL3	1
APEX2	1
PNKP	1
APLF	1
PARP3	1
ALKBH2	7
ALKBH3	7
MSH4	2
MSH5	2
PMS2P3	2
CETN2	3
DDB1	3
DDB2	3
GTF2H1	3
GTF2H3	3
GTF2H4	3
GTF2H5	3
CDK7	3
CCNH	3
MNAT1	3
LIG1	1, 3
ERCC8	3
UVSSA	3

XAB2	3
MMS19	3
DMC1	4
XRCC2	4
XRCC3	4
RAD54L	4
RAD54B	4
SHFM1	4
RBBP8	4
SLX1A	4
SLX1B	4
GEN1	4
FAAP20	5
DCLRE1C	6
NHEJ1	6
**Indirectly associated genomic stability maintenance genes**	
PAXIP1	
BLM	
MLL3	
CRIP1	
CDK12	
BAP1	
BARD1	
WRN	
BUB1	
CENPE	
ZW10	
TTK	

KNTC1
AURKB
POLB
POLH
POLQ
TDP1
TDP2
NUDT1
DUT
RRM2B
POLG
REV3L
MAD2L2
REV1
POLI
POLK
POLL
POLM
POLN
TREX1
TREX2
APTX
SPO11
ENDOV
UBE2A
UBE2B
RAD18
SHPRH

HLTF
RNF168
SPRTN
RNF8
RNF4
UBE2V2
UBE2N
H2AFX
CHAF1A
SETMAR
RECQL4
MPLKIP
DCLRE1A
DCLRE1B
PRPF19
RECQL
RECQL5
HELQ
RDM1
NABP2
ATRIP
MDC1
RAD1
RAD9A
HUS1
RAD17
TP53
TP53BP1

TOPBP1
CLK2
PER1
